# Rapid Microscopic Detection of *Bacillus anthracis* by Fluorescent Receptor Binding Proteins of Bacteriophages

**DOI:** 10.3390/microorganisms8060934

**Published:** 2020-06-21

**Authors:** Peter Braun, Immanuel Wolfschläger, Leonie Reetz, Lilia Bachstein, Ana Clara Jacinto, Carolina Tocantins, Johannes Poppe, Gregor Grass

**Affiliations:** Department of Bacteriology and Toxinology, Bundeswehr Institute of Microbiology (IMB), 80937 Munich, Germany; peter3braun@bundeswehr.org (P.B.); tgs_wolfschlaegeri@yahoo.de (I.W.); reetz.leonie@outlook.de (L.R.); lilia.bachstein@gmx.de (L.B.); aclarajacinto@gmail.com (A.C.J.); carolina.tocantins.23.3@gmail.com (C.T.); poppe.johannes0@hm.edu (J.P.)

**Keywords:** anthrax, *Bacillus anthracis*, bacteriophage, receptor binding protein, reporter fusions, detection assay

## Abstract

*Bacillus anthracis*, the etiological agent of anthrax disease, is typically diagnosed by immunological and molecular methods such as polymerase chain reaction (PCR). Alternatively, mass spectrometry techniques may aid in confirming the presence of the pathogen or its toxins. However, because of the close genetic relationship between *B. anthracis* and other members of the *Bacillus cereus sensu lato* group (such *as Bacillus cereus* or *Bacillus thuringiensis*) mis- or questionable identification occurs frequently. Also, bacteriophages such as phage gamma (which is highly specific for *B. anthracis*) have been in use for anthrax diagnostics for many decades. Here we employed host cell-specific receptor binding proteins (RBP) of (pro)-phages, also known as tail or head fibers, to develop a microscopy-based approach for the facile, rapid and unambiguous detection of *B. anthracis* cells. For this, the genes of (putative) RBP from *Bacillus* phages gamma, Wip1, AP50c and from lambdoid prophage 03 located on the chromosome of *B. anthracis* were selected. Respective phage genes were heterologously expressed in *Escherichia coli* and purified as fusions with fluorescent proteins. *B. anthracis* cells incubated with either of the reporter fusion proteins were successfully surface-labeled. Binding specificity was confirmed as RBP fusion proteins did not bind to most isolates of a panel of other *B. cereus s.l.* species or to more distantly related bacteria. Remarkably, RBP fusions detected encapsulated *B. anthracis* cells, thus RBP were able to penetrate the poly-γ-d-glutamate capsule of *B. anthracis*. From these results we anticipate this RBP-reporter assay may be useful for rapid confirmative identification of *B. anthracis*.

## 1. Introduction

*Bacillus anthracis* causing the zoonotic infectious disease anthrax in mammals and humans phylogenetically belongs to the *Bacillus cereus sensu lato* group of very closely related Firmicutes bacteria. The group comprises several familiar species, including *Bacillus cereus sensu stricto*, *Bacillus thuringiensis*, *Bacillus weihenstephanensis*, *Bacillus mycoides* and a variety of lesser characterized members [1]. Classical, culture-based techniques and classification upon phenotypic traits such as susceptibility against penicillin or lack of hemolysis are ambiguous and often fail to reliably differentiate *B. anthracis* from its close relatives. When comparing the 16S rRNA gene sequences, a very high degree of agreement can be observed among these species [2], thus far essentially disfavoring assays for species identification targeting these genetic elements. Similar challenges arise when using techniques such as multi locus sequence typing on members of the *B. cereus s.l*. group. In fact, most species of this group should be regarded as a single species [1]. However, some species carry characteristic virulence plasmids on which the genetic information for certain toxins is encoded. These include megaplasmid pCER270 for production of cereulide toxin in a clade of *B. cereus sensu stricto* strains [3] or plasmid pXO1 encoding a three-partite AB toxin from *B. anthracis* better known as lethal and edema toxin, respectively [4]. These phenotypic characteristics facilitate clinical differentiation, but do not always constitute reliable criteria for rapid identification of individual species. For example, virulence plasmids typical for *B. anthracis* (pXO1 and pXO2) can also be found in certain *B. cereus* isolates [1].

The crucial need for species identification without necessitating live bacteria is typically met by applying molecular methods such as polymerase chain reaction (PCR). For the identification of the tier 1 agent *B. anthracis*, chromosomal markers such as *PL3* [5], *dhp61* (BA5345) [6] or a nonsense mutation within the *plcR*-gene are frequently interrogated for [7]. Plasmids pXO1 and pXO2 are identified by virulence factor genes *pagA*, *lef*, *cya*, *capB* or *capC*, respectively [5,8,9]. In addition, immunological tests have been established which, due to their sensitivities to specific proteins, can not only detect antibodies after infection but also the pathogen’s antigens in the host blood such as the poly-γ-d-glutamic acids forming the bacterial capsule [10] or the toxin-subunit protective antigen (PA) during acute infection [11]. However, the challenge of species-specificity remains. Finally, a newer approach, matrix-assisted laser desorption ionization-time of flight mass spectrometry (MALDI-TOF MS), has proven successful because it facilitates rapid identification of difficult-to-identify pathogens such as *B. anthracis* [12].

In contrast to these assays which strongly rely on financial investments in equipment and consumables, the application of the classical bacteriophage (phage) plaque assay is both resource saving and easy to perform. As phages are viruses that only infect target bacteria, some phages have a very narrow host range accepting just a single species or even only a few strains within a species [13,14]. A number of virulent bacteriophages have been described in the literature that infect and multiply in *B. anthracis*. The most *B. anthracis*-specific phages can be assigned to the families Siphorviridae [15,16] and Tectiviridae [17,18] and always feature double-stranded DNA (dsDNA) as their genetic material. Brown et al. (1955) discovered the γ (gamma) phage which is a Siphovirus [16]. Phage γ has been introduced as a standard for plaque assay identification of *B. anthracis* [19,20,21], even though newer work has found a number of additional non-*B.-anthracis* strains susceptible to the phage [15]. Another *B. anthracis* specific phage named Wip1 (worm intestinal phage 1) is from the Tectiviridae family [18]. This phage was first isolated from the earthworm *Eisenia fetida* [18]. Schuch et al. (2010) compared Wip1 and γ phages for their host specificities towards *B. anthracis* and *B. cereus s. s.* strains. Remarkably, phage Wip1 achieved higher specificity than the γ phage [18,22]. Another Tectivirus phage that is very specific for *B. anthracis* is called AP50c [17]. This lytic phage was derived from temperate parental phage AP50t isolated from soil [23] and is genomically very similar to phage Wip1 but not identical [18]. Genome sequencing has revealed that the genome of *B. anthracis* contains four (inactive) prophages which have been named LambdaBa01-04 [24]. The presence of these prophages in a genome is also very specific for *B. anthracis*. This is especially true for LambdaBa01, 03 and 04 which were only found in strains of this species but not in close relatives of the *B. cereus s.l.* group [24].

The particular host specificity of phages is usually determined by receptor binding proteins (RBP) which enable the phage to recognize and bind to cell wall structures of the host bacterium [13,25]. In the above-mentioned specific “anthrax” phages, these receptor binding proteins (RBP) comprise the so-called tail (Siphorviridae) or head (Tectiviridae) fibers [25]. The RBP of phages Wip1 and γ were already provisionally characterized by in silico analysis and subsequent experimentation [18,26] but not yet the RBP of phage AP50c or of prophage LambdaBa03. The structural make-up of the typically homotrimeric RBP is similar in many phages [27,28]. RBP feature two critical domains: at the *N*-terminus, the RBP is anchored to the phage (head or tail) while the recognition and binding domain is located at the *C*-terminus of the protein. This binding domain can either confer narrow or broad specificity. The corresponding surface structures of the bacteria (i.e., the receptors), which are responsible for recognition and adsorption of the phage or its RBP can be quite different, including such diverse entities as polysaccharides, teichoic acids, structural or capsule proteins [27,28]. Davison et al. (2005) showed that for the binding of the γ phage, the receptor protein GamR of *B. anthracis* is essential [29]. The RBP of γ phage was identified as the product of the *gp14* gene on the phage genome [26]. For phage Wip1 the receptor of *B. anthracis* has not yet been unambiguously identified but it has been proposed from earlier work that the surface layer protein Sap (surface array protein) is involved in binding by the RBP either directly or indirectly [18]. The CsaB protein, a cell-surface anchoring protein, was found to be required for phage AP50c adsorption [30]. Because Sap is anchored by CsaB, Sap is the likely receptor for the *B. anthracis* specific phage AP50c [31], yet no indication of the RBP involved was given. From these previous works we further characterized *Bacillus* (pro)-phage RBP and developed tools to be used in routine DNA-independent, fluorescence microscopic rapid identification of the highly pathogenic bacterium *B. anthracis*.

## 2. Materials and Methods

### 2.1. Bacterial Culture and Inactivation

Unless specified differently, *B. anthracis* strains and other Bacilli were cultivated at 37 °C on tryptic soy agar plates (TSA, Merck KGaA, Darmstadt, Germany) or in 250 mL baffled flasks containing 50 mL tryptic soy broth (TSB, Merck KGaA) with shaking at 110 rpm. All risk group 3 (RG-3) *B. anthracis* strains were grown in the biosafety level 3 (BSL-3) laboratory at the Bundeswehr Institute of Microbiology (IMB) and then chemically inactivated before further use [32]. Inactivation of RG 2 strains for subsequent RBP reporter tests was carried out by pelleting 1 mL of a bacterial culture at 5000× *g* for 3 min and resuspending the cell pellet in aqueous peracetic acid solution (2% Terralin PAA, Schülke & Mayr GmbH, Norderstedt, Germany) or 4% paraformaldehyde (Merck KGaA) and incubating at room temperature for 30 or 60 min, respectively. For heat inactivation another sample was resuspended in PBS and incubated at 98 °C for 30 min (with heated lid cover). After inactivation, all samples were washed twice with PBS. For cultivation of encapsulated *B. anthracis* cells, a fresh colony from a TSA plate was used to inoculate 5 mL of Luria Bertani (LB) broth (Merck KGaA) containing 0.8% NaHCO_3_ in cell culture flaks (Nunc EasYFlask 25 cm^2^; ThermoFisher Scientific, Darmstadt, Germany) followed by incubation with 10% CO_2_ atmosphere at 37 °C for 4 h or overnight. *Escherichia coli* cultures were grown in LB broth or on Luria Bertani (LB)-agar (Merck KGaA) with 20 µg/mL of gentamycin and 100 µg/mL carbenicillin (Carl Roth, Karlsruhe, Germany) where required.

### 2.2. Spore Preparation

Sporulation and subsequent spore purification of *B. anthracis* and other Bacilli was done as previously described [33] with slight modifications. A colony of a fresh overnight culture of *B. anthracis* (or other Bacilli) was used to inoculate 50 mL sporulation medium containing 0.8% nutrient broth (Merck KGaA) amended with 0.05 mM MnCl_2_, 0.7 mM CaCl_2_ and 1.0 mM MgCl_2_ [34] in 500 mL baffled flasks. After incubation at 37 °C and 110 rpm shaking for 72 h, Tween 80 was added to a final concentration of 3% and the culture incubated for another 24 h. The culture was transferred to a 50 mL centrifugation tube and harvested by centrifugation at 2000× *g* and 20 °C for 10 min. The supernatant was discarded and the pellet washed twice with 25 mL 3% Tween 80 and further incubated on a rotary shaker at 150 rpm for 24 h. Purity of spore suspensions was checked by phase contrast microscopy and spores were harvested by centrifugation (2000× *g* and 20 °C for 10 min) when purity was above 95% (fewer than 5% vegetative cells present). If purity was less than 95%, spores were washed again with 25 mL 3% Tween 80 and incubated for another 24 h until purity was sufficient. Finally, the spore pellet was resuspended in 3 mL ice-cold ultrapure H_2_O and stored at 4 °C until further use. Spore preparations reached concentrations of up to 10^9^ spores per mL.

### 2.3. Isolation of DNA

DNA from Bacilli and bacteriophages was isolated using MasterPure™ Gram Positive DNA Purification kit (Lucigen, Middleton, WI, USA) and DNA concentrations quantified using the Qubit dsDNA HS Assay Kit (ThermoFisher Scientific) according to the manufacturers’ protocols. DNA preparations were stored at −20 °C until further use.

### 2.4. Cloning of RBP-Fusion Constructs

For construction of genetic *RBP-mCherry*-fusions, first the *mCherry* open reading frame from plasmid mCherry-pBAD (mCherry-pBAD was a gift from Davidson, Shaner and Tsien, University of California at San Diego, La Jolla, CA, USA; Addgene plasmid #54630 (company, Watertown, MA, USA; http://n2t.net/addgene:54630; RRID:Addgene_54630; [35]) was PCR amplified creating overhangs containing restriction sites for Esp3I for cloning into pASG-IBA105 expression plasmid downstream of the *twin-strep-tag* epitope sequence. Primer overhangs also introduced recognition sites for restriction enzymes SalI, EcoRI and BsrGI as well as XhoI, PstI and BsiWI upstream and downstream of the *mCherry* gene, respectively, for subsequent insertion of RBP genes. Primer sequences are listed in Table 1. One-step Esp3I digestion and ligation was carried out using StarGate Direct transfer cloning System (IBA GmbH, Göttingen, Germany) as described in the manufacturer’s protocol and plasmids were transformed into NEB Turbo cells (New England Biolabs GmbH, Frankfurt am Main, Germany). Clones were confirmed by Sanger sequencing (Eurofins Genomics Germany GmbH, Ebersberg, Germany) of their recombinant pASG-mCherry plasmids using primers flanking the insert. Next, for generation of *mCherry-RBP* fusions, respective RBP genes were PCR amplified from purified DNA generating XhoI and BsiWI overhangs and were digested with XhoI and BsiWI alongside pASG-mCherry. After ligation of the fragments, constructs were transformed and plasmid sequences of clones checked as described above.

### 2.5. Expression and Purification of Strep-Tagged mCherry-RBP Fusion Reporters

The pASG-mCherry::RBP plasmids were transformed into *E. coli* ArcticExpress cells (Agilent Technologies Inc., Waldbronn, Germany). Single colonies harboring recombinant plasmids with fusion constructs were used to inoculate 5 mL of LB medium with gentamycin and carbenicillin in a 50 mL centrifugation tube. After overnight incubation at 37 °C with shaking at 150 rpm, 400 µL of the culture were added to a 1000 mL baffled flask containing 200 mL prewarmed LB medium and incubated at 30 °C with shaking at 110 rpm until the optical density (OD_600_) of the culture reached 0.6–0.8. Temperature was decreased to 12 °C and gene expression derepressed with a final concentration of 0.2 ng/mL anhydrotetracycline (AHT; IBA GmbH, Göttingen, Germany) for 24 h. The culture was harvested by centrifugation and the cell pellet resuspended in 50 mL lysis buffer containing 100 mM Tris-HCl, pH 8.0, 1 mM EDTA, 150 mM NaCl, 40 µg/mL lysozyme and 1% Halt Protease-Inhibitor Cocktail, EDTA-free (ThermoFisher Scientific). Mechanical lysis was carried out using a French press system (Emulsiflex-C3; Avestin Europe GmbH, Mannheim, Germany) and lysate was then centrifuged at 10,000× *g*, at 4 °C for 10 min and filtered through a 0.45 µm pore size syringe filter.

For subsequent affinity chromatography using the Äkta pure system (GE Healthcare Life Science, Munich, Germany), the cleared lysate was loaded onto 1 mL Streptactin XT columns (IBA GmbH, Göttingen, Germany), washed with 20 mL buffer W (100 mM Tris-HCl, pH 8.0, 1 mM EDTA, 150 mM NaCl) and the protein was eluted with buffer BXT (buffer W containing 50 mM biotin). After dialysis against a 1000-fold volume of HEPES buffer (50 mM HEPES, 50 mM NaCl, 5 mM EDTA, pH 7.5) using SnakeSkin 10K MWCO dialysis membrane (ThermoFisher Scientific), protein concentrations were measured with Pierce BCA Protein Assay Kit (ThermoFisher Scientific). Next, Amicon Ultra 15 Centrifugal Filters 10K MWCO (Merck KGaA) were used to adjust protein concentrations to 1 mg/mL and protein aliquots were directly stored at −80 °C until further use or first amended with 50% glycerol (final concentration) as cryo-protectant and kept at −20 °C for testing in RBP-fusion reporter assays.

### 2.6. SDS-PAGE and Western Blot

Protein samples were mixed with 10× NuPAGE Sample Reducing Agent (ThermoFisher Scientific) and 4× NuPAGE LDS Sample Buffer (ThermoFisher Scientific), denatured at 95 °C for 5 min and applied to a polyacrylamide gel (Novex NuPAGE 4–12% Bis-Tris protein-gel, 1.0 mm, 10-well; ThermoFisher Scientific) using a mini gel tank (ThermoFisher Scientific). SDS-PAGE was performed at 200 V for 60 min with MOPS running buffer (ThermoFisher Scientific). Then the proteins were transferred onto a 0.45 µm pore size nitrocellulose membrane (ThermoFisher Scientific) at 30 V for 75 min via semidry blotting (Novex Semi-Dry Blotter, ThermoFisher Scientific) in NuPAGE transfer buffer. Pierce Reversible Protein Stain Kit (ThermoFisher Scientific) was used to stain whole blotted protein before detection of Strep-tagged proteins, which was carried out using Strep-MAB-Classic (HRP conjugate, IBA GmbH) based chemiluminescence detection and Clarity Western ECL substrate (Bio-Rad Laboratories, Munich, Germany) according to the manufacturers’ protocols. A ChemiDoc MP imaging system (Bio-Rad Laboratories) and image Lab 5.2 software (Bio-Rad Laboratories) were used for documentation.

### 2.7. RBP Testing for Binding to Host Cells

An overnight culture of *B. anthracis* or other Bacilli was used to inoculate 50 mL of fresh TSB in a 250 mL baffled shaking flask to an optical density (OD_600_) of 0.05 and the culture was incubated at 37 °C and 110 rpm. For growth phase experiments starting from spores, 10^7^ spores were used to inoculate 50 mL of brain heart infusion (BHI, Merck KGaA) broth containing 10% fetal bovine serum (Merck KGaA). At different time points, OD_600_ was measured and a sample taken equivalent to 100 µL of an OD_600_ of 1 (for non-germinated spores as inoculum a volume of 1 mL of the inoculated culture was used as first sample at T_0_). Treatment was the same for inactivated or encapsulated *B. anthracis* cells. Samples were pelleted by centrifugation at 5000× *g* for 2 min in 1.5 mL centrifugation tubes, resuspended in 100 µL of Ringer-HEPES buffer (50 mM HEPES, 1.5 mM CaCl_2_, 1.5 mM KCl, 100 mM NaCl, 0.6 mM NaHCO_3_, pH 7.4) and mixed with 5 µg of purified RBP fusions. After 5 min incubation at room temperature, cells were washed once with Ringer-HEPES (5000× *g* for 2 min) and 3 µL were transferred into a well of a chamber slide with lid (µ-slide 8 Well, Ibidi GmbH, Martinsried, Germany). When encapsulated cells were tested, samples were mixed with an equal volume of ink prior to transfer to the chamber slide. For proper microscopic analysis of cells, suspensions were covered with a 1 mm thick agar-agar pad serving as a coverslip (1% molten agar-agar solidified between two microscopy slides). Samples were analyzed for cells emitting mCherry signal (extinction: 587 nm, emission: 610 nm) from bound RBP fusions using Axio Observer Z1 700 Confocal Laser Scanning Microscope (Carl Zeiss, Oberkochen, Germany).

## 3. Results

### 3.1. Cloning of Phage RBP Genes and Production of Recombinant RBP-Fusion Reporters in E. coli

When initiating this work we performed sequence similarity database searches in order to identify relatives of RBP from phages γ (protein Gp14) and Wip1 (P23) [36] and did protein sequence alignments in order to identify possible RBP of phage AP50c (Figure S1). We recognized that a hypothetical protein, BA4079, very similar to RBPγ was encoded on the *B. anthracis* chromosome located within previously identified prophage LambdaBa03 [24]. Protein alignment of BA4079 with RBPγ (Gp14) revealed amino acid (aa) identities of 83.0% (similarity 89.0%) across the entire length (500 aa) of the alignment (Figure S1). These values increased to 95.2% and 98.4% when only the *C*-terminal section of the proteins, comprising a continuous stretch without gaps of 374 aa were reanalyzed. The second information gained from this database search relevant for the study at hand, was that there is not any (hypothetical) protein encoded on the genome of phage AP50c that has significant similarity to the one identified from phage Wip1. However, we recognized a corresponding (hypothetical) polypeptide to Wip1 P24 in phage AP50c. P24 from phage Wip1 was found to be required for RBP_Wip_ (P23) activity [18]. The respective gene encoding the hypothetical AP50c protein P29 is located directly downstream of a gene for yet another hypothetical protein, P28, without any relatives in the database. When P28 (phage AP50c) was aligned with P23 (phage Wip1) the identity score was low, only 32 out of 151 aa (21.2%) with a similarity of 36.4% but featuring 52 gap positions (Figure S1). Remarkably, the first seven aa residues of both polypeptides (MGLKKPS) were a perfect match. Thus, by genomic position and the short identical stretch to P23, we selected putative protein P28 to be further studied as RBP candidate of phage AP50c.

**Figure 1:**
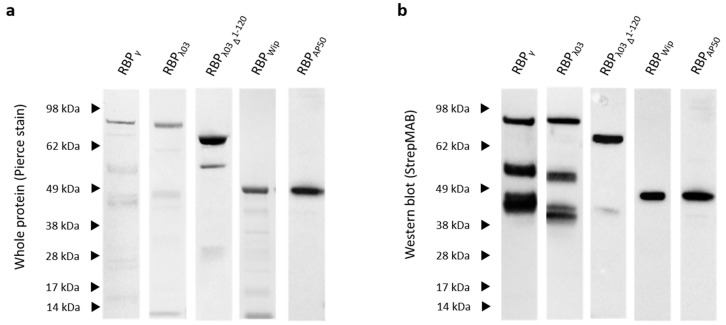
Western blot of heterologously produced RBP-fusion reporter proteins. Affinity purified proteins were subjected to SDS-PAGE, stained (Pierce stain) after transfer onto a nitrocellulose membrane (**a**) and the TST epitope detected using a HRP-conjugated TST-antibody (StrepMAB) (**b**). Expected sizes of RBP mCherry reporters: RBP_γ_ 88 kDa, RBP_λ03_ 88 kDa, RBP_λ03Δ1-120_ 74 kDa, RBP_Wip_ 44 kDa, RBP_AP50_ 46 kDa. Letters indicate the size-positions of the protein size marker (SeeBlue Plus2 prestained, ThermoFisher Scientific, Darmstadt, Germany).

As a basis of a versatile expression plasmid for production of fluorescent reporter fusions, a plasmid chassis was constructed. For this, the PCR-amplified gene of mCherry was cloned in *E. coli* using expression vector pASG-IBA105, which contains a *twin-strep-tag*–encoding sequence (*tst*), resulting in pASG-mCherry. The previously identified RBP genes from phages Gamma and Wip1, as well as putative RBP genes from phage AP50c and prophage λ03 were PCR-amplified from genomic DNA and inserted in-frame downstream to the fluorescent protein gene in vector pASG-mCherry, to yield a set of plasmids of the following composition; pASG::*tst*::*mCherry*::*RBP_γ/Wip/AP50/λ03_*. In case of constructs harboring RBP from phages Wip1 and AP50c the gene downstream of the RBP gene on the phage genome was cloned as a transcriptional fusion to the RBP gene. This is because in a previous study the necessity of this adventitious protein for RBP function has been demonstrated [18]. Thus, if not stated otherwise for RBP_Wip/AP50_ the term RBP comprises two polypeptides in our study (P23+P24 for phage Wip1 and P28+P29 for phage AP50c). Nevertheless, we also included production of P23 or P28, respectively, alone in plasmids pASG::*tst*::*mCherry*::*P23_Wip_/P28_AP50_*. Also, we included 5′-truncated versions of the RBP_λ03_ gene. Aiming at improving solubility of the corresponding protein, coding regions of the following peptides were cloned as well: RBP_λ03Δ1-120_, RBP_λ03Δ1-139_, RBP_λ03Δ1-316_, RBP_λ03Δ1-342_. All fusion proteins were produced in *E. coli* ArcticExpress, as other *E. coli* expression strains tested were found to produce insoluble proteins mostly incorporated into inclusion bodies. Sizes of fusion proteins purified by affinity chromatography were confirmed by Western blotting as shown in Figure 1 and Figure S2.

Protein yield of RBPγ was low, most of the protein was found as insoluble inclusion bodies. Proteins could also be obtained for RBP_λ03Δ 1-139_, RBP_λ03Δ1-316_, and RBP_λ03Δ1-342_ (Figure S2) as well as for P23 and P28 alone. Truncated RBP_λ03Δ1-120_ was soluble and gave higher protein yields than full-length RBP_λ03_ (Figure 1). A minor degradation signal was detected for this RBP_λ03Δ1-120_ by protein staining (Figure 1a). This byproduct lacked a TST epitope because it was not visible after TST detection (Figure 1b). A faint smaller-sized degradation product of RBP_λ03Δ1-120_ featuring the TST was observed by Western blotting (Figure 1b). More prominently degraded TST-labeled RBP reporters were detected for RBP_λ03_ and RBPγ (Figure 1b).

### 3.2. (Pro)-Phage RBP Bind to Bacillus anthracis Cells

**Figure 2:**
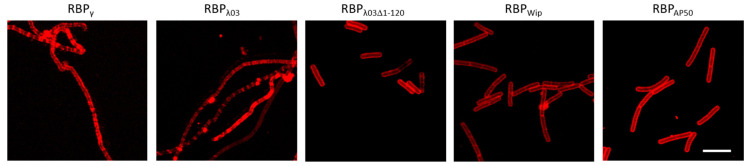
Representative fluorescent microscopy detection of *B. anthracis* cells using RBP reporters. Cultures of *B. anthracis* CDC 1014 were grown for 2–3 h, washed with HEPES-Ringer-buffer, mixed with RBP, washed again to remove unbound RBP and subjected to fluorescence microscopy. Shown are mCherry-RBP reporters of phage Gamma (RBP_γ_), prophage LambdaBa03 (RBP_λ03Δ_ and truncated RBP_λ03Δ1-120_), phage Wip1 (RBP_Wip_) and phage AP50 (RBP_AP50_). Scale bar: 10 µm.

The RBP fusion proteins were next tested for their abilities to bind to *B. anthracis* cells, especially with regard to putative RBP_AP50_ P28(+P29), as the RBP of phage AP50c has not been identified thus far. A second emphasis was on putative RBP_λ03_ and its truncated derivatives. For testing of RBP binding, 2–3 h old vegetative cultures of *B. anthracis* CDC 1014 were used. The RBP_λ03_ reporter showed binding to *B. anthracis* as microscopically detectable fluorescence and cell surfaces were visibly labeled (Figure 2). Of the deliberately truncated RBP_λ03_ only RBP_λ03Δ1-120_ was able to bind to cells (Figure 2). Binding to cell surfaces of this truncated RBP_λ03Δ1-120_ was stronger than that of the full-length parent RBP_λ03_ (Figure 2). Related RBPγ reporter also yielded signals, however, similar to full length RBP_λ03_ most of the protein was found in insoluble inclusion bodies. Thus, further testing of low-yield RBPγ and RBP_λ03_ as well as of non-binding derivatives RBP_λ03Δ1-139_, RBP_λ03Δ1-316_ and RBP_λ03Δ1-342_ was abandoned in favor of the other RBP reporters including RBP_λ03Δ1-120_.

Fluorescent labeling of *B. anthracis* cells was also achieved for RBP_AP50_ P28(+P29) (Figure 2), which supported our initial hypothesis that P28(+P29) is the actual RBP of phage AP50c. When AP50c P28 was tested by itself (and also when Wip1 P23 was tested by itself) only a very weak binding signal was observed (Figure S3) and thus, P28 and P23 alone were also abandoned for further testing. These results suggest P29 playing a pivotal role for proper function of P28 as RBP. Also, Figure 2 supports our hypothesis that locus *BA4079* of prophage Lambda03 encodes for a RBP (RBP_λ03_) and that its truncated derivative RBP_λ03; Δ1-120_ functions as a *B. anthracis* reporter.

### 3.3. Binding of (Pro)-Phage RBP to B. anthracis Cells Is Growth Phase Dependent

**Figure 3:**
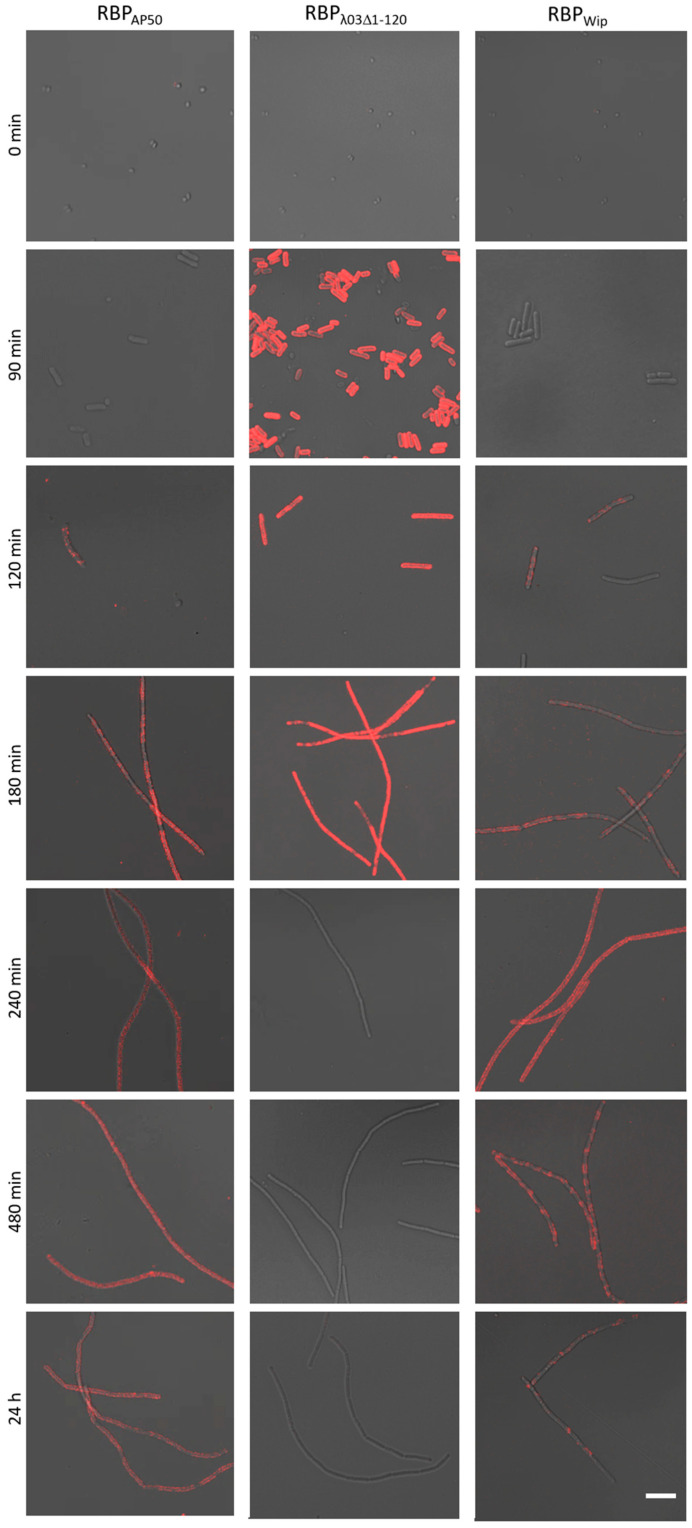
Binding of red-fluorescent RBP reporters to *B. anthracis* cells at different time points during culture growth phases. Tested were germinating *B. anthracis* Sterne spores over a period of 24 h. Representative time points are shown for binding of the three reporters RBP_AP50_, RBP_λ03Δ1-120_ and RBP_Wip_ recorded in merged light and fluorescence channels. Scale bar: 10 µm.

During the initial RBP reporter binding experiments we observed that RBP fusion proteins exhibited variations in their binding patterns. We reasoned this is most likely dependent on the host’s growth phase since we did not use synchronized *B. anthracis* cultures in initial binding experiments. In particular, RBP_λ03Δ1-120_ fusion proteins showed declining binding signals on cell surfaces of *B. anthracis* CDC 1014 or Sterne the longer cultures grew (Figure 2 and Figure 3). To investigate this observation in more detail, growth experiments were carried out for *B. anthracis* CDC 1014 or Sterne cultures starting from spores. Since spores need time to germinate, differences in RBP binding patterns should occur as a function of cultivation time. RBP binding was monitored from culture samples taken at intervals of typically 30 min during a period of 0 to 8 h including a final 24 h sample.

From the micrographs depicted in Figure 3 (time point T_0_ min), it can be seen that none of the RBP fusion reporters of (pro)-phages AP50c, LambdaBa03 or Wip1 showed any detectable fluorescence signals when tested on non-germinated spores. It is thus likely that RBP do not bind to spores under the conditions tested. This finding was corroborated by incubating these RBP reporters with spores of *B. anthracis* Sterne and *B. cereus* strains 10987 and 4342 as well as *B. thuringiensis* 10792.

Conversely, all RBP reporters produced significant fluorescent signals on cell surfaces of germinated *B. anthracis* spores at the latest after 120 min, with the RBP_λ03; Δ1-120_ fusion being the only one that already showed binding after 90 min. The RBP_λ03Δ1-120_ reporter reached maximum binding signal after 120 min, whereupon the signal remained strong, decreasing after 180 min and was no longer detectable after 240 min. However, this complete lack of binding in later growth phases did not occur in each growth experiment conducted. If, for example, vegetative cells of an overnight culture were inoculated instead of spores, the signal was also retained in later phases and even after 24 h, which was certainly due to the unsynchronized cell division.

For the RBP_AP50_ reporter, the first fluorescence signal on germinating spores was detected after 120 min, which continuously intensified and reached its peak after about 180 to 240 min, whereupon it remained constant for several hours and only became slightly weaker between 8 and 24 h.

A similar fluorescence signal pattern was observed when RBP_Wip_ was tested. The strongest binding signal was scored 180 to 240 min after germination was initiated. In the further course the signal became significantly weaker between 8 and 24 h (Figure 3) and featured incompletely distributed, patchy fluorescence signals on the cell surfaces (e.g., RBP_Wip_ at 480 min; also compare Figure 2). This “tiger stripes” pattern also appeared yet more weakly on ageing cells labeled with RBP_AP50_ or RBP_λ03Δ1-120_, respectively.

**Figure 4:**
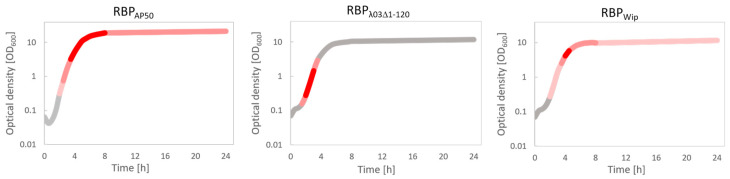
Binding of RBP reporters to *B. anthracis* cells varies between RBP of different phages but yields strongest signals during logarithmic growth phase of host cells. Growth experiments were started from germinating *B. anthracis* Sterne spores and the growth phase was derived from recordings in optical cell densities at 600 nm (OD_600_). Binding strengths of reporters RBP_AP50_, RBP_λ03Δ1-120_ or RBP_Wip_ are indicated by colored sections of the growth curves: grey = no binding, light red = weak RBP binding, i.e., either only sporadic binding and/or binding to only a few areas of the cell surface. red: distinct RBP binding (easily recognizable binding to the majority of all cells). dark red = very strong RBP binding ((almost) all cells exhibit very intense fluorescence signals).

In order to show the temporal RBP binding pattern on *B. anthracis* cells in a semi-quantitative manner, we next correlated RBP reporter signal strength with *B. anthracis* growth phases during growth experiments (growth curves). Analysis showed that all three RBP reporters feature maximum binding to host cells via fluorescence during logarithmic growth phase of *B. anthracis* cultures (Figure 4). The earliest response exhibited the RBP_λ03Δ1-120_ reporter from early to mid-logarithmic growth phase (Figure 4), the latest, RBP_Wip_, peaking near the end of logarithmic growth (Figure 4). In contrast, the RBP_AP50_ reporter yielded measurable signals from the mid-logarithmic growth phase, climaxing at late logarithmic-phase to early stationary phase yet remained clearly detectable until the end of the experiments (Figure 4). Thus, it appears that the RBP_AP50_ reporter was the most versatile for this RBP recognized cells in the widest range of growth phases, except spores and freshly germinated spores (<2 h) (compare Figure 3, e.g., RBP_Wip_ at 480 min).

Next, we compared these results with that of not-synchronized cultures featuring cells of different growth phases. In contrast to that of synchronized cultures, the results here were quite erratic, as would be expected. Some patterns, however, emerged. Binding of the RBP_Wip_ reporter was maximum at the start of the cultures and after 3 to 5 h. RBP_λ03Δ1-120_ recognized cells best between 1 and 2.5 h. Binding of RBP_AP50_ was most constant; weaker signals were obtained only around 3 h, 7 h and after 24 h. In contrast to synchronized cultures, weak fluorescence signals could be obtained at any time using any of the three RBP reporters on non-synchronized cultures.

### 3.4. Inactivated Cells of B. anthracis Can Be Labeled With (Pro)-Phage RBP Reporters

Often times it is not possible to work with live cultures of *B. anthracis* e.g., in field laboratory settings lacking required equipment or in the absence of a fluorescence microscope in BSL-3 facilities. Also, mindful of laboratory work safety, we were curious whether it was possible to use the RBP reporters on inactivated *B. anthracis* cells. To test this, we evaluated different in-house validated *B. anthracis* inactivation regimens for suitability of subsequent RBP reporter binding on inactivated cells of *B. anthracis* strains Sterne or CDC1014. Cultures were inactivated either by heat, formaldehyde or peracetic acid.

**Figure 5:**
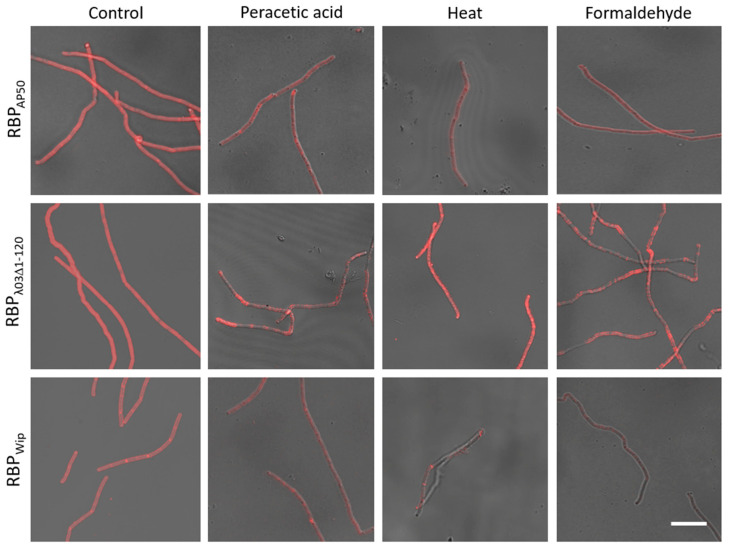
Binding of RBP reporters to inactivated cells of *B. anthracis* CDC1014. Cells were either inactivated by heat, formaldehyde or peracetic acid treatment. Inactivated cells or live control cells (from the same culture) were incubated with RBP reporters (RBP_AP50_, RBP_λ03Δ1-120_ or RBP_Wip_) and subjected to fluorescence microscopy. Representative micrographs recorded as merged light and fluorescence channels are shown. Scale bar: 10 µm.

Cells inactivated by heat yielded strong fluorescence signals upon binding of the RBP_AP50_ and RBP_λ03Δ1-120_ reporters similar to fluorescence levels of non-inactivated cells. Conversely, heat-inactivated cells were only poorly labeled by the RBP_Wip_ reporter (Figure 5). Similarly, formaldehyde-inactivation did not prevent the binding of the RBP_AP50_ and RBP_λ03Δ1-120_ reporters but the RBP_Wip_ reporter did not bind. In contrast, inactivation with peracetic acid yielded fluorescence signals for all three RBP reporters upon binding to inactivated cells, however, of lower intensities than the controls (Figure 5). Nevertheless, this line of experiments made possible the further use of inactivated *B. anthracis* cells and of inactivated cells of other pathogenic Bacilli.

Thus, we then tested binding of the RBP reporters on inactivated cells of fully virulent *B. anthracis* isolates of risk group 3 (RG 3) from our collection. These strains were of diverse phylogenetic composition from all three major branches A, B and C [37]. RBP reporter binding was done on overnight cultures and on fresh, 4 h old cultures inoculated thereof. Cultures of RG 2 strains were inoculated under the same conditions as controls as some of these strains have been used for the experiments described above. All RG 3 strains were successfully labeled by the three RBP reporters yet with different signal strengths (Table 2).

Most strains yielded strong fluorescent signals for any of the three reporters, yet cells of the C-branch isolate A1074 were labeled less efficiently. Also, cells of B-branch strains seemed to be accessible to the three RBP reporters, though binding of RBP_λ03Δ1-120_ was more efficient than binding of RBP_AP50_ or RBP_Wip_. A similar pattern was observed for A-branch strain Vollum (Table 2).

### 3.5. Encapsulated Cells of Bacillus anthracis Can Be Labeled with (Pro)-Phage RBP Reporters

**Figure 6:**
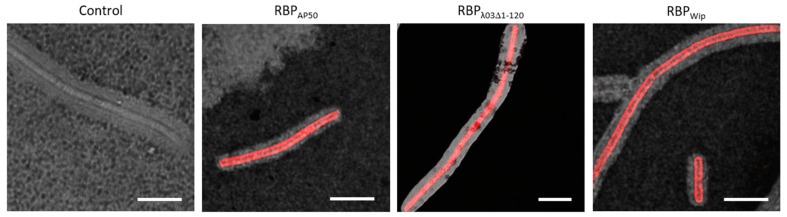
Binding of RBP reporters to encapsulated cells of *B. anthracis*. Fresh cultures of *B. anthracis* strain Ames inoculated from overnight cultures were grown for 4 h under capsule-inducing conditions and then inactivated by peracetic acid treatment. Cells were incubated with RBP reporters (RBP_AP50_, RBP_λ03Δ1-120_ or RBP_Wip_) or no RBP (control) and subjected to fluorescence microscopy in the presence of negative-staining ink. Representative micrographs were recorded as merged light and fluorescence channels. Scale bars: 10 µm.

When grown in host mammals, *B. anthracis* produces a poly-d-glutamyl capsule for averting the host’s immune response. We tested thus to which extend this capsule would hinder penetration and binding of RBP reporters to *B. anthracis* cells. For this, pXO2 (capsule) positive *B. anthracis* strain Ames and the other six RG-3 strains from Table 2 were grown under capsule inducing conditions, after 4 h of growth in fresh inducing culture, cells were inactivated using peracetic acid and negative-stained with ink. The three RBP reporters were added and samples subjected to fluorescence microscopy. Figure 6 shows that the capsule did not prevent cell labeling by the three RBP reporters. All samples gave strong fluorescence signals, clearly showing binding of the RBP reporters amidst the capsule and the bacterial cell as exemplified by the Ames strain (Figure 6). Even the largest of the three RBP reporters, RBP_λ03Δ1-120_, was able to label encapsulated cells.

Care had to be taken when adjusting the ink concentration, otherwise capsule visualization by negative staining with black ink eclipsed fluorescence signals noticeably. Notwithstanding this caveat, this line of experiments clearly demonstrated that all three RBP reporters, RBP_AP50_, RBP_λ03Δ1-120_ and RBP_Wip_ were capable of labeling encapsulated cells of *B. anthracis*.

### 3.6. Binding of (Pro)-Phage RBP Is Specific to B. anthracis Cells

**Figure 7:**
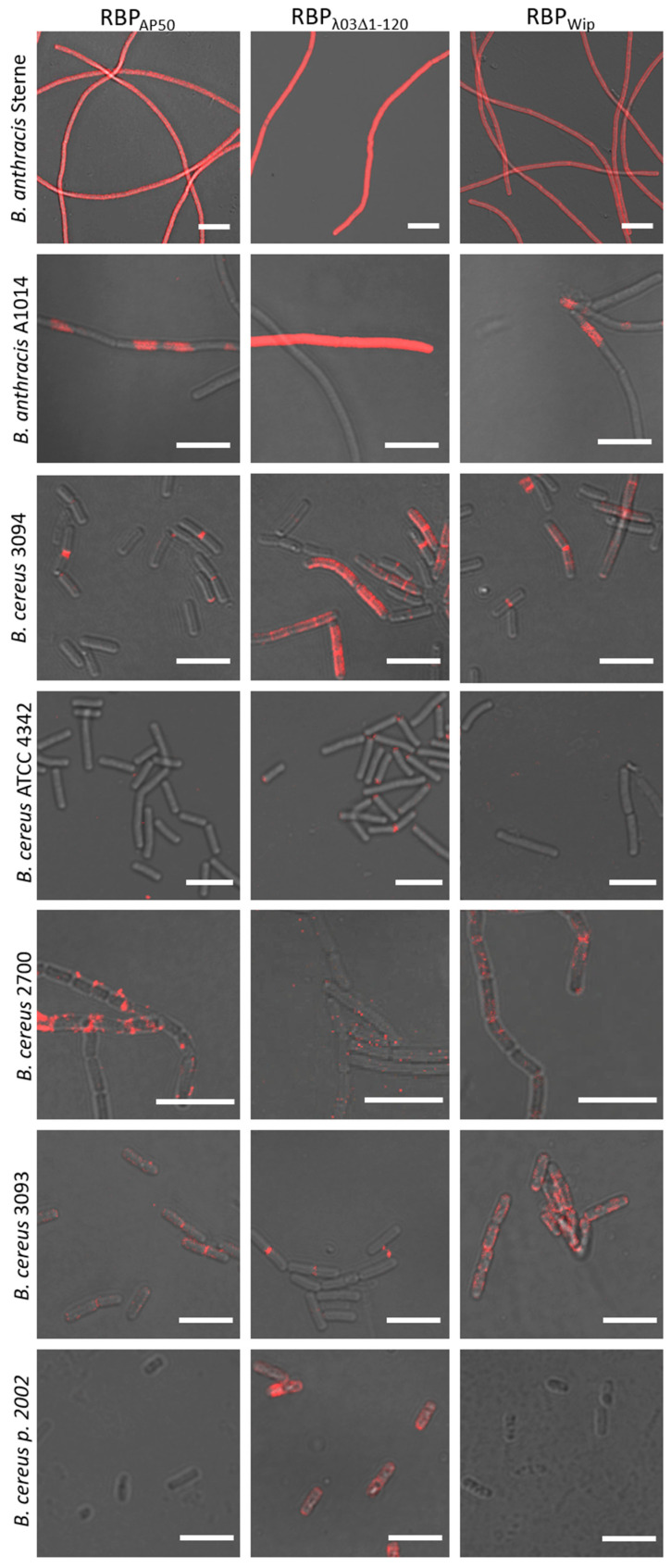
Binding of RBP reporters to cells of non-*anthracis* bacilli. Fresh cultures inoculated from overnight cultures of representative strains from Table 3 were grown for 2 (for labeling with RBP_λ03Δ1-120_) or 4 h (for labeling with RBP_AP50_ or RBP_Wip_), incubated with RBP reporters (RBP_AP50_, RBP_λ03Δ1-120_ or RBP_Wip_) and subjected to fluorescence microscopy. Representative micrographs were recorded as merged light and red fluorescence channel. Cells of *B. anthracis* Sterne and A1014 served as genuine positive examples. Scale bars: 10 µm.

Next, we determined RBP reporter binding to a panel of *Bacillus* strains closely related to *B. anthracis*. Of 56 non-*anthracis Bacillus* ssp. tested, most (42%) did not bind any of the three RBP reporters at all and a small number (12%), only very weakly (Table 3; Figure 7). Three strains (*B. cereus* 3094, *B. paranthracis* 2002 and *B. weihenstephanensis* B0293) were significantly labeled by the RBP_λ03Δ1-120_ reporter, yet clearly yielding a weaker signal than *B. anthracis* host cells, even distinct from signals of cells of rare *B. anthracis* C-branch strain A1074 (Figure 7). Cells of a single one of these strains (*B. cereus* 3094) was also markedly labeled by RBP_AP50_ and RBP_Wip_ reporters. Again higher fluorescence signals upon RBP reporter binding were observed when *B. anthracis* cells (even the few colored cells of C-branch strain A1074 were more uniformly labeled) were used as positive control hosts (Table 3; Figure 7). Thus, from analysis of Table 3 and mindful of the results depicted in Figure 7 we suggest specificities of the RBP reporters may be as high as 98% (1 false positive out of 56 non-*anthracis* Bacilli) for RBP_AP50_ (*B. cereus* 3093) and RBP_Wip_ (*B. cereus* 2700) or 95% (three false positives out of 56 non-*anthracis* Bacilli) for RBP_λ03Δ1-120_ (*B. cereus* 3094, *B. weihenstephanensis* B0293 and *B. paranthracis* 2002).

## 4. Discussion

The confirmatory specific identification of *B. anthracis* is often achieved by means of antigen–antibody interaction, be it in the form of enzyme-linked immunosorbent assays (ELISA) [38], lateral flow assays [39,40] or by the use of fluorescently labeled antibodies in microscopy [41] (further alternative detection techniques for *B. anthracis* are reviewed in a contribution to the special issue “An Update on Anthrax” of Microorganisms [42]). The direct fluorescent-antibody (DFA) assay [41] may be seen as being related to the study at hand insofar as both methods take advantage of fluorescence reporters for detecting presumptive *B. anthracis* cultures and thus helping confirming the identity of this notorious biothreat agent. In contrast to RBP fused to fluorescent protein for visual labeling of target cells, recognition via DFA was achieved using two fluorescent dye-labeled monoclonal antibodies, one specifically directed to the galactose/*N*-acetylglucosamine polysaccharide cell wall antigen, the other one recognizing the capsule antigen. Only when these two antibody reporters were combined, the DFA assay reached high specificity. Of 230 *B. anthracis* isolates tested 227 were positive (99% specificity) and 56 of 56 non-*anthracis Bacillus* strains were found to be negative. This DFA assay is fast, taking less than 1 h for completion [41], thus requiring only moderately more time than the RBP reporter assay described here (see graphical abstract for a visualization of the timescale of the assay).

The use of RBP (and other phage-derived proteins) for detection and identification of bacteria is not a new methodology; its utility has been extensively reviewed [13,43,44]. Depending on the specificities of utilized phage proteins, RBP can even be used for the identification and separation of different isolates of the same species according to different O-antigens on the surface of *Listeria monocytogenes* cells [45]. The situation might be viewed as similar to the situation of *B. anthracis* within the *B. cereus s.l.* group of bacteria. Taking into account the very close genetic relationship of *B. anthracis* to *B. cereus*, *B. anthracis* may also be considered a subspecies within the *B. cereus s.l.* group [46]. Thus, our RBP reporters would detect a subspecies as well, in this case *B. anthracis*.

In contrast to the canonical *B. anthracis* typing phage γ [21], phage Wip1 showed no binding to cells of an untypical strain of *B. cereus* (strain ATCC 4342) [18,22]. This result correlates with our findings regarding lack of recognition of RBP_Wip_ to cells of this *B. cereus* isolate. Kan et al. (2013) in their infection and adsorption tests showed affinity of the Wip1 phage for the *B. cereus* CDC 2000032805 strain [47], which is also a host for the γ phage [18]. Since this *B. cereus* CDC 2000032805 strain was not available to us, RBP reporter binding to this strain could not be tested and thus we were not able to assess if RBP_Wip_ (or RBP_AP50_) would merely show marginal binding as did a small group of the other Bacilli tested (Table 3) or whether this untypical host would be recognized as efficiently as *B. anthracis*. However, recognition of phage Wip1 RBP proteins P23(+P24) to cells of strain CDC2000032805 was shown previously as a patchy fluorescence pattern [18]. Thus, strain CDC2000032805 must be added to list of *B. cereus s.l*. strains able to be partly recognized by RBP_Wip_.

Phage AP50c infected 111 of 115 tested *B. anthracis* strains (~97%) except for e.g., a Sap mutant of Sterne strain and none of 100 *B. cereus sensu lato* strains [17]. Remarkably, in the same study two out of three different Vollum derivatives also were insensitive to phage AP50c. With today’s knowledge on the receptor of phage AP50 [31], these are derivatives that have likely lost their S-layers. Later, two additional *B. cereus* strains were found to be sensitive to phage AP50c, *B. cereus* RS438 (CDC2000032805) and *B. cereus* RS756 (better known as E33L ZK; Zebra Killer [48]), with efficiencies of plating reduced by about one third compared to a *B. anthracis* Sterne derivative host. Two additional strains (*B. thuringiensis* serovar *pulsiensis* BGSC 4CC1 and *B. thuringiensis* serovar *monterrey* BGSC 4AJ1) allowed adsorption of the phage but not propagation. In contrast, *B. cereus* ATCC 4342 sensitive to phage γ, was insensitive to phage AP50c [31]. In the same study, Sap was identified as likely receptor of phage AP50 [31]. This finding was supported by a parallel study in which by analysis of mutants that failed to support AP50 propagation, the CsaB protein was found to be required for phage AP50 host recognition and adsorption. CsaB is required for cell-surface anchoring of the S-layer and thus including Sap [30]. Our and earlier observations [18] of “tiger stripes” binding patterns of RBP reporters (Figure 3) that depend on host cell growth phases support earlier notions [18]. In their thorough characterization of the phage Wip1 genome and several protein functions, the authors suspected a temporal change in S-layer proteins Sap to EA1 (extractable antigen 1 encoded by the *eag* gene) when cultures of *B. anthracis* exit from logarithmic into stationary phase [49,50] and that this was the underlying reason for diminished binding of RBP P23 in their study [18].

Likely, the Sap protein may also be the reason why *B. cereus* strains 2700, 3093 and 3094 cross-reacted weakly with our AP50 and Wip reporters (Table 3). Some *B. cereus* strains possess Sap proteins that have a high similarity to Sap of *B. anthracis* [31]. Likewise, binding of RBP_λ03Δ1-120_ reporter to several *B. cereus* strains (Table 3) may be based on similarities of their GamR receptor (i.e., the receptor of phage γ and likely also the receptor for RBP_λ03_) with that of *B. anthracis* [29]. Because RBP reporter binding is dependent on the presence of the cognate receptors (Sap or GamR, respectively) on the bacterial host cell surface, recognition is not dependent on the presence or absence of *B. anthracis* virulence plasmids (pXO1 and pXO2). Thus, *B. cereus* strains harboring such plasmids [1] cannot *per se* be expected to be labeled by the RBP reporters. Conversely, the rather weak binding of RBP reporters to cells of rare *B. anthracis* C-branch strain A1074 may be linked to the overall poor growth of this strain in our hands on both solidified and liquid media.

Interestingly, phage Bam35, a Tectivirus of *B. thuringiensis* genomically closely related to phages Wip1 and AP50 does not encode for proteins related to P23 or P28, respectively [51]. Attempts to identify the RBP of this Bam35 phage have thus far been unsuccessful. The protein encoded by a gene occupying the same location on the phage Bam35 genome (P29) as P23/P24 (of phage Wip1) [18] or P28/P29 (of phage AP50) did not bind to host cells, though this protein is very likely positioned on the surface of Bam35 virons [52]. Instead this phage seems to utilize different means of host cell recognition. Experiments with peptidoglycan isolated from Bacilli and *E. coli* demonstrated that *N*-acetyl-muramic acid in the bacterial cell wall is required for binding of phage Bam35 [52]. Thus, even in genomically closely related *Bacillus* Tectivirus phages it is not always that straightforward to identify (i) the phage RBP and (ii) the cellular receptor recognized by this RBP.

Though we abandoned early experiments with phage γ RBP (Gp14) reporters because of protein yield and solubility issues, this protein nevertheless bound to cells of *B. cereus* ATCC 4342 (Figure S4). This result agrees with earlier results on a different host cell wall binding protein, the endolysin PlyG of phage γ [53]. This PlyG is produced from phage-infected cells right before new virions are to be released from the dying host cell. PlyG depolymerizes the peptidoglycan from within after PlyG is transported across the cytoplasmic membrane. However, PlyG can also act from without, digesting the *B. anthracis* cell wall when added to growing cells [53]. The authors found PlyG to be highly specific for *B. anthracis* cells; only two non-B. *anthracis* Bacilli were targeted: strain *B. cereus* RSVF1 (identical to strain 4342 [53]) and *B. cereus* ATCC 10987. These two were among the isolates our RBP_λ03Δ1-120_ reporter recognized weakly (Table 3; Figure 7). Though endolysins are typically investigated for as alternative antimicrobial compounds [54], the terminal cell wall binding domain (CBD) of PlyG was later used for *B. anthracis* detection as a bioprobe [55]. The bioprobe assay was tested for specificity on two *B. anthracis* and 17 *Bacillus* spp. strains, however, atypical *B. cereus* strains such as strain 4342 were not included. Notably, and in concurrence with our results using RBP reporters for detection of *B. anthracis*, PlyG-CDB was able to detect encapsulated cells, however, spores were not detected unless germination was induced first. While this PlyG-CBD detection seemed to be specific, the detection assay took a couple of hours to complete [55]. This PlyG-based detection assay was later further developed by shortening the PlyG-CDB down to 20, 15 or 10 aa residues and by including attached fluorescent Qdots for microscopic analysis. Remarkably, even the shortest derivative was able to bind to *B. anthracis* Sterne cells but not to cells of three other *B. cereus s.l.* strains tested [56]. However, similar to our RBP_λ03_ reporter, cells of *B. cereus* strain 4342 were also labeled. Further, while our new RBP reporter assays take about 10 min to perform from harvesting cell cultures to fluorescence microscopy (see graphical abstract for details), PlyG-Qdots detection took at least 3 h because two 90 min incubation steps are required [56].

In order to accelerate phage-based detection and identification of *B. anthracis* such methods have also seen significant improvements. One is combining phage-amplification in its natural host coupled with phage nucleic acid amplification by PCR [57]. This assay can be expected to be as specific as the classical plaque assay for the oligonucleotide primers used were tested for specificity against a DNA negative panel. A short phage propagation period is followed by signal (DNA) amplification by real time PCR. This approach shortened the total assay time to about 5 h (including 4 h of growth of host and phage) [57]. The host-mediated phage amplification/PCR amplification hybrid identification approach has recently seen methodological improvements. In an improved technique named phage-mediated molecular detection (PMMD) a short incubation period of bacterial host culture (*Staphylococcus aureus* or *B. anthracis*) with a species-specific phage is followed by RNA extraction and reverse transcription PCR (RT-PCR) on specific phage transcripts [58]. The authors thus took advantage of the high number (relative to DNA) of phage RNA molecules produced per infected cell, which can be expected to far exceed the number of nascent phage DNA genomes. Indeed, the concentration of phage RNA after host infection was sufficient for the generation of strong signals. In this assay *B. anthracis* was grown prior to RT-PCR for 3 h without phage followed by an infection phase of about 13 min and RNA-preparation. A further advantage of this technique is that it can be coupled to antibiotic susceptibility testing [58]. Nevertheless, in contrast to the new RBP reporter assay, *B. anthracis* detection by PMMD requires growth of live bacteria not always possible, especially in field settings [59], whereas RBP reporters introduced here, may be also used on inactivated cells if required.

## 5. Conclusions

In this work, we developed RBP proteins of several (pro)-phages of *B. anthracis* into microscopy-based detection tools. In doing so we identified two new RBP from phage AP50c and chromosomally integrated prophage LambdaBa03. Detection can be achieved within about 10 min when live cells of *B. anthracis* are used, yet the assay also works very well on inactivated and on encapsulated cells. The assay is very specific, especially in the case of the RBP reporters constructed from RBP of phages AP50c and Wip1, while RBP_λ03Δ1-120_ exhibited a slightly broader host range basically following the specificities of their parental phages. Of note, however, our RBP reporter assay is a qualitative rather than a quantitative detection method requiring a fluorescence microscope. Because of its rapidity and specificity we envision this RBP reporter assay to be able to supplant the original phage based plaque-assay for confirmative pathogen identification in laboratories with access to fluorescence microscopy.

## Figures and Tables

**Table 1:** Primers used for cloning.

Oligonucleotide	Sequence (5′–3′)
BA4079 forward	AAACTCGAGATGAGTTCTTTTTCATTTAATGGGGAAC
BA4079 reverse	AAACGTACGTCTGTATCTCTCCCTATAACTGATTGTTG
BA4079λ03_Δ1-120_ forward	AAACTCGAGATGAGTTCTTTTTCATTTAATGGGGAAC
gp14 forward	AAACTCGAGTTGGGGAAACTTAGTTTTACTTTTAATAATATTAG
gp14 reverse	AAACGTACGTCTATATCTCTCCCTATAACTGATTGTTGC
p23 forward	AAACTCGAGATGGGACTTAAGAAACCTGCGG
p23 reverse	AAACGTACGTTCATAAGCAACCCACGGTTG
p23+p24 reverse	AAACGTACGCATTCCTCCTAGTAATATATCGTTAATTGCAC
p28 forward	AAACTCGAGATGGGACTGAAAAAACCTAGCGG
p28 reverse	AAACGTACGGAATGGTTTTTCCGCTTCCTCTTTTAC
p28+p29 reverse	AAACGTACGCATTCCTCCTAATAGAATATCGTTAATTGTAC
mCherry forward	AGCGCGTCTCCAATGGTCGACGGTGAATTCGGCTGTACAGTTAGTAAAGGAGAAGAAAATAACATGGC
mCherry reverse	AGCGCGTCTCCTCCCCGTACGGCCCTGCAGACCCTCGAGTTTGTATAGTTCATCCATGCCACCAG

Restriction endonuclease recognition sites are underscored.

**Table 2:** Labeling of RG-3 *B. anthracis* cells with RBP reporters ^1^.

Cultures of Peracetic Acid-Inactivated *B. anthracis* Strains RG-3 (RG-2)	Phylogenetic Group	RBP_AP50_	RBP_λ03Δ1-120_	RBP_Wip_
(CDC 1014)	A.Br.WNA	+++	+++	+++
(Sterne 34F2)	A.Br.001/002	+++	+++	+++
Vollum	A.Br.Vollum	++	+++	++
188678-1	A.Br.Aust94	+++	+++	+++
Ames	A.Br.Ames	+++	+++	+++
A0777	A.Br.WNA	++	+++	+++
BF-1	B.Br.CNEVA	++	+++	++
SA020	B.Br.Kruger	++	+++	++
A1074	C.Br.	+	++	+

^1^ Cultures were tested for binding of RBP reporters after 4 h of growth and overnight culture with similar results. (+): weak RBP binding, i.e., either only sporadic binding and/or binding to only a few areas of the cell surface; (++): distinct RBP binding, i.e., easily detectable binding to the majority of cells; (+++): very distinct RBP binding, i.e., almost all or all cells with very intense fluorescence signal.

**Table 3:** Labeling of *Bacillus* spp. cells with RBP reporters ^1^.

Species Strain	RBP_AP50_	RBP_λ03Δ1-120_	RBP_Wip_
*B. anthracis* Sterne 34F2	+++	+++	+++
*B. anthracis* A1074	+	++	+
*B. cereus* 3094	+	++	+
*B. cereus* 2700, 3093	+	−*	+
*B. cereus* ATCC 706, ATCC 4342 ^2^, ATCC 10987, DSM 2302, 2832	−	+	–
*B. cereus* ATCC 312	−	−*	−
*B. cereus* ATCC 14579, ATCC 2787, ATCC 3301, DSM 345, 288, 1356, 2690, 2698, 2815, 2830, 2856, 2866, 2868, 2892, 2893, 2894, 2895, 2896, 2897, 2899, 2900, 2901, 2902, 2903, 2904, 2905, 3045, 3068, 3080, 3090, 3092, 3095, 3096, 3097, 3098, 3109	−	−	−
*B. thuringiensis* ATCC 10792, DSM 2046	−	+	+
*B. thuringiensis* ATCC 336, ATCC 33679	−	−*	−
*B. thuringiensis* Berliner 1915, sv. Tolworthi	−	−	−
*B. paranthracis* 2002	−	++	−
*B. weihenstephanensis* B0293	−	++	−
*B. mycoides* NCTC 2603, B 732	−	−	−
*B. megaterium* ATCC 14581, MS941	−	−	−
*B. subtilis* ATCC 6091	−	−	−

^1^ Cultures were tested for binding of RBP reporters after 2 (RBP_AP50_ and RBP_Wip_) and 4 h (RBP_λ03Δ1-120_) of growth. (−): no RBP binding; (−*) few weakly labeled cells; (+): weak RBP binding, i.e., either only sporadic binding and/or binding to only a few areas of the cell surface; (++): distinct RBP binding, i.e., easily detectable binding to the majority of cells; (+++): very distinct RBP binding, i.e., almost all or all cells with very intense fluorescence signal. **^2^** is a known host of phage γ [18].

## Supplementary Materials

The following are available online at https://www.mdpi.com/2076-2607/8/6/934/s1, Figure S1: Protein sequence alignments of RBP and accessory proteins of *Bacillus anthracis* phages Wip1 AP50 and prophage lambdaBa03; Figure S2: Pierce stain of heterologously produced additional or truncated RBP reporter fusions; Figure S3: Binding of different fluorescent RBP reporter fusions to *B. anthracis* cells; Figure S4: Binding of RBPγ reporter to *B. cereus* ATCC4342 cells.
