# Supplementary material for: Rapid Microscopic Detection of Bacillus anthracis by Fluorescent Receptor Binding Proteins of Bacteriophages
Source: Microorganisms. 2020 Jun 21;8(6):934. doi: 10.3390/microorganisms8060934 (PMC7356292; doi:10.3390/microorganisms8060934)
Supplement: Supplementary file 1 [file microorganisms-08-00934-s001.pdf]

Supplementary material for: Rapid microscopic detection of *Bacillus anthracis* by fluorescent receptor binding proteins of bacteriophages

Peter Braun[1], Immanuel Wolfschläger[1], Leonie Reetz[1], Lilia Bachstein[1], Ana Clara Jacinto[1], Carolina Tocantins[1], Johannes Poppe[1], and Gregor Grass[1*]

[1]Bundeswehr Institute of Microbiology (IMB), Munich, Germany; peter3braun@bundeswehr.org (P.B.); tgs_wolfschlaegeri@yahoo.de (I.W.); reetz.leonie@outlook.de (L.R.); lilia.bachstein@gmx.de (L.B.); aclarajacinto@gmail.com (A.C.J.); carolina.tocantins.23.3@gmail.com (C.T.S.); poppe.johannes0@hm.edu (J.P.); gregorgrass@bundeswehr.org (G.G.)

(a)
AP50 (RBP$_{AP50}$ protein P28)
Wip1 (RBP$_{Wip}$ protein P23)

```
Matrix: EBLOSUM62; gap_penalty: 10.0; extend_penalty: 0.5; length: 151;
identity: 32/151 (21.2%); similarity:55/151 (36.4%); gaps: 52/151 (34.4%);
score: 56.5

AP50               1 MGLKKPS----------GAPFMSAQGESFVTIESQKSATLEINNLSFTPN     40
                     ||||||:          |.||......|:|         :|:..|||.|:
Wip1               1 MGLKKPAVGGKRVAKGIGKPFQPQGSASWV---------VEVRGLSFKPS     41

AP50              41 MIVIR-CETQYGEVYQMTYHPFQMIYGINVTRGV---NVDKNRPTYLSND     86
                     ::..: .::|....|     |:: :..:.:.|..   .:|::....::||
Wip1              42 VVATKPKKSQIDADY-----PYR-VGTVGIARTAFEPPLDEDLLNMINND     85

AP50              87 ---SYG-LLTPSAKGFNAKLTPDTDIFRGSALFKWKAYYFPSVKEEAEKP    132
                        |.| ::|....||...|....:       ..|.||.
Wip1              86 GDYSVGRVVTFYDDGFKIYLDKGNE-------QPWVAYE-----------    117

AP50             133 F    133

Wip1             118 -    117
```

(b)
AP50 (protein P29)
Wip1 (protein P24)

```
Matrix: EBLOSUM62; gap_penalty: 10.0; extend_penalty: 0.5; length: 118;
identity: 60/118 (50.8%); similarity: 85/118 (72.0%); gaps: 0/118 (0.0%);
score: 324.0

AP50               1 MFVPLRIYYDKKTGLIIQYTGNFQDNNMIQAPTIEDDFTSYQSLNERVKE     50
                     |:|..|:|::|.||::::.||.|:|:.:...||:::|...|..|.|||.:
Wip1               1 MYVNKRVYFEKDTGIVVMVTGGFRDDWLHSHPTVQEDMAKYSVLAERVPD     50

AP50              51 TVGVIELEQNQYKEELYKATNVTVDVKTGQLMFDFTPIVKKEIEEKKTLE    100
                     |:.::||::..|.||..||.:..|||||..::|||||..|||:|||||.|
Wip1              51 TLSMLELKEGTYDEEFSKARSFKVDVKTNTIVFDFTPEDKKEVEEKKTPE    100

AP50             101 QRITLVESTINDILLGGM    118
                     .|:|:|||.|||||||||
Wip1             101 HRVTMVESAINDILLGGM    118
```

(c)

Lambda03 (RBP$_{\lambda 03\Delta 1\text{-}120}$)

Gamma (RBP$_{\gamma}$)

Matrix: EBLOSUM62; gap_penalty: 10.0; extend_penalty: 0.5; identity: 415/500 (83.0%); similarity: 445/500 (89.0%);gaps: 7/500 (1.4%); score: 2205.0

```
Lambda03          1 ------mssfsfngerksyihiergwkrpiwaplrrnflsvpsypgarll     44
                          ..||:||..||.||.:..|.|||.|||::|..:.||...||.||
Gamma             1 mvstlgklsftfnnirkdyiqmlvgrkrpswapvkrrlvrvphragalll     50

Lambda03         45 ntqtemrvfsvpvgiiapsgv-dmkilsediaswlitdqpkelifdtepd     93
                    ||:||.|...||:.|.|...: |::.|.||:|.||.|:||.|||||.|.|
Gamma            51 nteteerridvplvikakkdmadlqklkedladwlyteqpaelifddeld    100

Lambda03         94 rtylavvdeefdadefveigqgnlkficpmpyklgkinthkftqewstet    143
                    ||||:::|...|.||.|..|:|.:.|:|||||||||||||||||||||||
Gamma           101 rtylslidgsvdldeivnrgkgvitfvcpmpyklgkinthkftqewstet    150

Lambda03        144 tsfftnkgsveapaliemtvkkpstfldvwfgeyphnrdyfrigypltve    193
                    ||:|||||||||||||||||||||||||||||||||||||||||||||||
Gamma           151 tsyftnkgsveapaliemtvkkpstfldvwfgeyphnrdyfrigypltve    200

Lambda03        194 ettvqerervmwdematpigwtpvtgqfddmkgtgsfksrggyalycedy    243
                    ||||||||||||||||||||||||||||::||||||||||||:|||||||
Gamma           201 ettvqerervmwdematpigwtpvtgqfeemkgtgsfksrgghalycedy    250

Lambda03        244 gkevgfygaiakknipggplqdfemeawmtlksknigemgrvevllldea    293
                    |||.||||||||||||||||||||||||:|||||||.||||||||||||.
Gamma           251 gketgfygaiakknipggplqdfemeawvtlksknisemgrvevllldet    300

Lambda03        294 snvvarinmndlyataeitrahmkignsgtpnsfrklvdtsgyysttfnq    343
                    |||::||||||||||||||||||.||||||||||||||||||:|||||||
Gamma           301 snvisrinmndlyataeitrahmtignsgtpnsfrklvdtsgfysttfnq    350

Lambda03        344 frgrlriarrgkvwsvyvakfidgtekdgaslverwidetgnpmterkia    393
                    ||||||||||||||||||||||||||||||||||||||||||||||||||
Gamma           351 frgrlriarrgkvwsvyvakfidgtekdgaslverwidetgnpmterkia    400

Lambda03        394 qvmiaickwdnhepvneiqiddlkfwkvnkvpsnaqpyifdtgdkividt    443
                    ||||||||||||:|:||:||||||.|||||||||||||||||||||||||
Gamma           401 qvmiaickwdnhqpinemqiddlkiwkvnkvpsnaqpyifdtgdkividt    450

Lambda03        444 ekslvtingknainikeifsnfpivirgenridimppdvnatisyreryr    493
                    |||||||||:.|||||||||||||||||||||||||||||||||||||||
Gamma           451 ekslvtingekainikeifsnfpivirgenridimppdvnatisyreryr    500
```

Supplementary Figure S1: Protein sequence alignments of RBP and accessory proteins of *Bacillus anthracis* phages Wip1 AP50 and prophage LambdaBa03. Alignments of RBP proteins and their putative chaperons. Shown are pair-wise amino acid residue sequence alignments (single letter code) of (a) RBP$_{AP50}$ (protein P28) with RBP$_{Wip}$ (protein P23); of (b) the putative RBP chaperons P28 of phage Wip1 with P23 of phage AP50c, and of (c) RBP$_{\lambda 03\Delta 1\text{-}120}$ (protein BA4079) with RBP$_{\gamma}$ (protein Gp14). Highlighted in green are starting positions of N-terminally truncated derivatives of RBP$_{\lambda 03}$. Protein sequence alignments were performed with EMBOSS Needle using standard parameters [1].

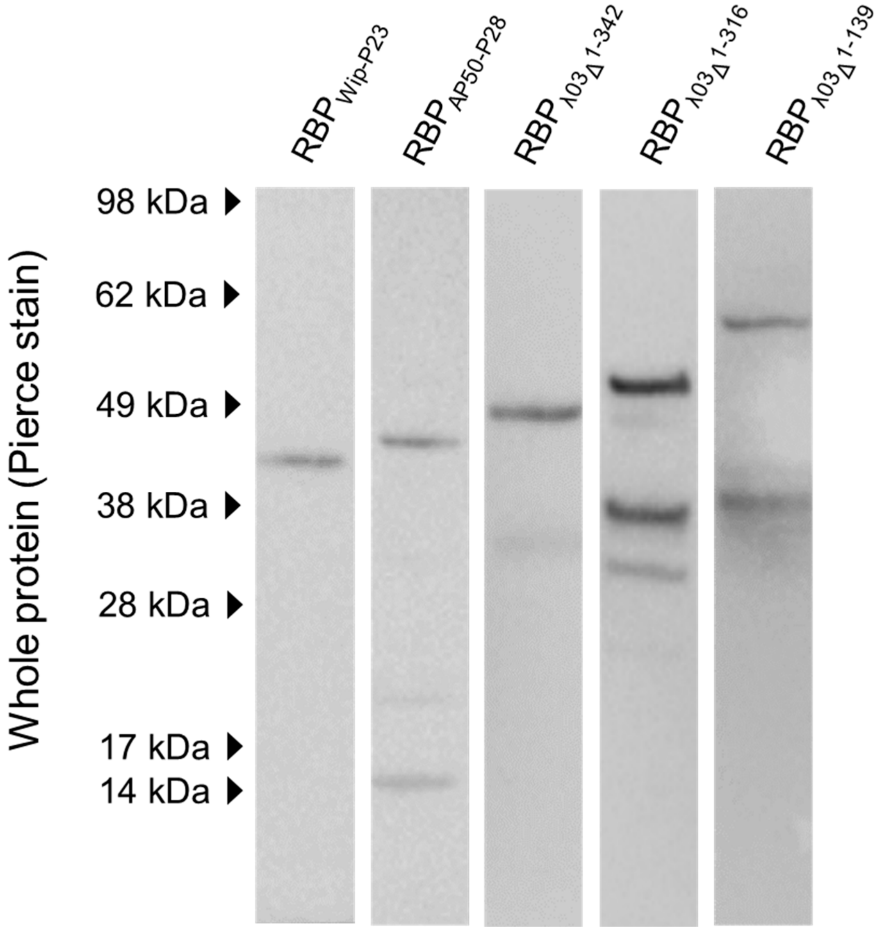

Supplementary Figure S2: Pierce stain of heterologously produced additional or truncated RBP reporter fusions. SDS-PAGE with subsequent staining (Pierce stain) was carried out after transfer of proteins onto a nitrocellulose membrane. Expected sizes of mCherry RBP fusions are: $RBP_{Wip\text{-}P23}$ 44 kDa, $RBP_{AP50c\text{-}P28}$ 46 kDa, $RBP_{\lambda03\Delta1\text{-}342}$ 49 kDa, $RBP_{\lambda03\Delta1\text{-}316}$ 52 kDa, $RBP_{\lambda03\Delta1\text{-}139}$ 74 kDa. Letters indicate the size positions of the protein size marker (SeeBlue Plus2 prestained (ThermoFisher Scientific, Darmstadt, Germany).

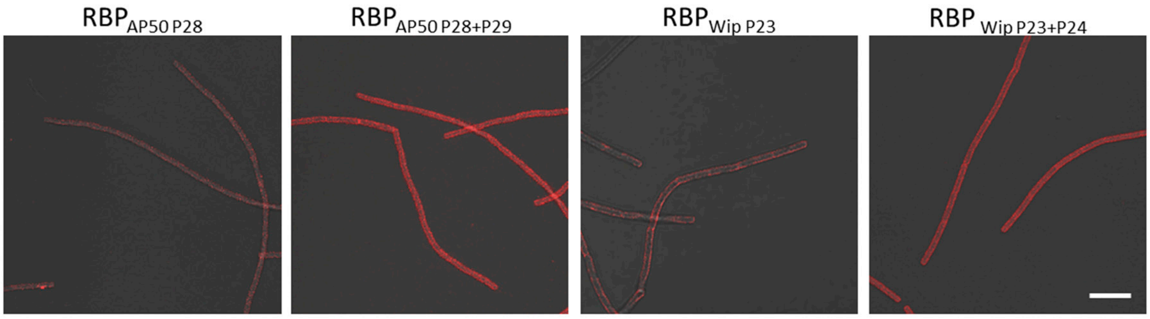

Supplementary Figure S3: Binding of different fluorescent RBP reporter fusions to *B. anthracis* cells. Cells of *B. anthracis* Sterne were incubated with RBP reporters ($RBP_{AP50\ P28}$, $RBP_{AP50\ P28\ +P29}$, $RBP_{Wip\ P23}$ or $RBP_{Wip\ P23+P24}$) and subjected to fluorescence microscopy. Representative micrographs were recorded as merged light and fluorescence channels. Scale bar: 10 µm.

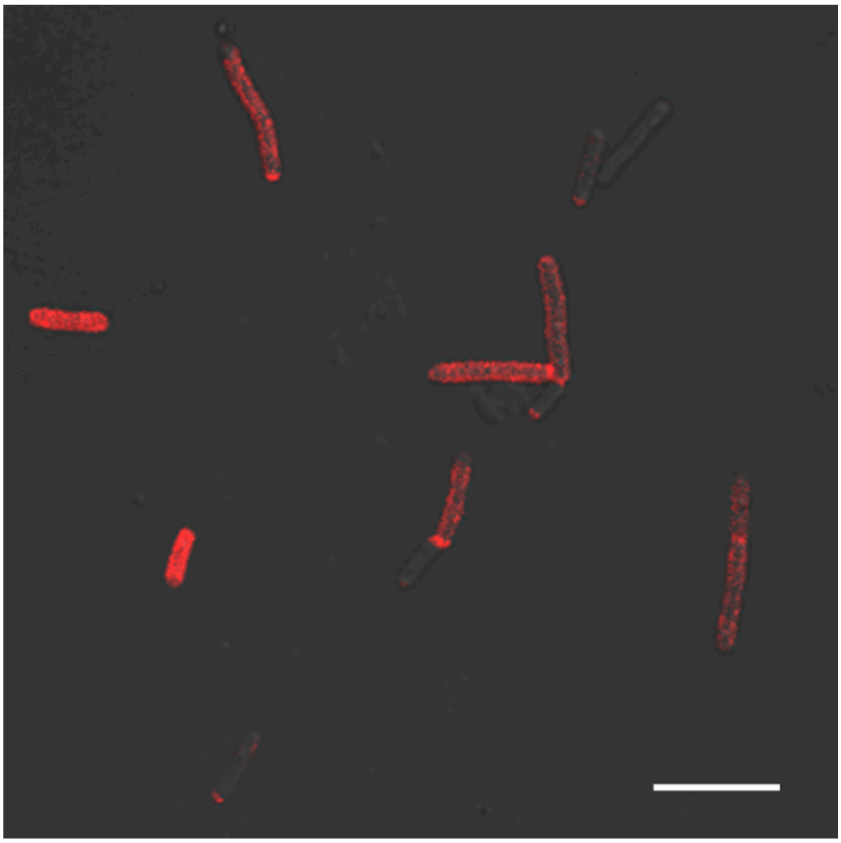

Supplementary Figure S4: Binding of RBP$_\gamma$ reporter to *B. cereus* ATCC4342 cells. *B. cereus* ATCC4342 was grown for 2-3 h, washed with HEPES-Ringer-buffer, mixed with RBP$_\gamma$ reporter, washed again to remove unbound RBP and subjected to fluorescence and bright-field microscopy. Scale bar: 10 µm.

Reference

1 Needleman, S. B. & Wunsch, C. D. *J Mol Biol* **48**, 443-453, doi:10.1016/0022-2836(70)90057-4 (1970).
